# Artifact Mimicking Mechanical Valve Dysfunction on Intraoperative Transesophageal Echocardiography Following Redo Mitral Valve Replacement

**DOI:** 10.7759/cureus.102096

**Published:** 2026-01-22

**Authors:** McAndrew Merlini, Keijiro Mitube, Suguru Tatsuki, Gentaku Hama, Ryuji Koushima

**Affiliations:** 1 Cardiovascular Surgery, Sapporo Heart Center, Sapporo, JPN

**Keywords:** cardiac reoperation, echocardiographic artifact, intraoperative fluoroscopy, intraoperative imaging, mechanical heart valve, mirror image artifact, mitral valve replacement, stuck valve, transesophageal echocardiography, valve dysfunction diagnosis

## Abstract

Intraoperative transesophageal echocardiography (TEE) is vital for assessing prosthetic valve function, but imaging artifacts can lead to misdiagnosis. We describe a case of a 54-year-old man who underwent redo mitral valve replacement with a mechanical prosthesis, in which intraoperative TEE suggested prosthetic leaflet immobility. This prompted surgical re-exploration, which revealed normal valve function. Despite resection of posterior leaflet remnants that may have caused leaflet immobility, the abnormal findings persisted on TEE but were ultimately shown to be artifacts on intraoperative fluoroscopy. This case highlights the potential for TEE artifacts to mimic mechanical valve dysfunction. Multimodal imaging is critical for accurate diagnosis and avoiding unnecessary re-intervention.

## Introduction

Transesophageal echocardiography (TEE) is a cornerstone of intraoperative assessment during cardiac surgery. According to data from the STS Adult Cardiac Surgery Database, TEE was used in 73% of all open cardiac surgeries [1]. Its use was associated with significantly better outcomes, including lower 30-day mortality, lower stroke incidence, and fewer reoperations [2].

TEE provides real-time intraoperative monitoring that can confirm or alter diagnoses, guide device and catheter placement, and assess procedural success. It plays a vital role in detecting complications such as valve dysfunction and residual defects, making it essential for surgical decision-making [3].

In mitral valve surgery, TEE is especially valuable for assessing prosthetic valve function [4]. However, despite its high-resolution imaging capabilities, both 2D and 3D TEE are susceptible to artifacts [5]. Evaluating prosthetic valves poses unique challenges due to refraction, acoustic shadowing, reverberation, and mirror artifacts generated by the prosthesis's reflective surfaces [6]. Misinterpretation of these artifacts may result in unnecessary surgical reintervention, prolonged cardiopulmonary bypass (CPB) time, and increased morbidity.

Here, we report a rare case of intraoperative TEE artifact mimicking a stuck mechanical mitral valve leaflet, ultimately clarified with fluoroscopic imaging.

## Case presentation

Patient background

A 54-year-old man with a history of hypertension and hyperlipidemia had previously undergone two mitral valve plasty procedures for degenerative mitral regurgitation, the initial surgery approximately five years earlier, followed by a redo procedure three years prior to the current presentation. Preoperative transthoracic and TEE demonstrated severe mitral regurgitation with preserved left ventricular function. He was classified as New York Heart Association Class II. As part of the preoperative evaluation, routine preoperative computed tomography imaging was performed.

Operative details

The patient underwent a third mitral valve operation. General anesthesia was induced uneventfully. CPB was established via ascending aorta and bicaval cannulation. The aorta was cross-clamped, and myocardial arrest was achieved using antegrade cardioplegia. A transseptal approach was used. Intraoperative findings revealed thickened posterior leaflets and ruptured artificial chordae from prior repairs. The anterior mitral leaflet was excised, while its chordae tendineae were preserved and anchored to the commissural region to maintain subvalvular continuity. The posterior leaflet and its associated chordae were preserved intact, in accordance with standard practice to support left ventricular geometry and function. A 31-mm mechanical mitral valve (SJM Masters Series, Abbott Medical, St. Paul, MN, USA) was implanted using interrupted pledgeted sutures. Total CPB and aortic cross-clamp times were 364 minutes and 218 minutes, respectively.

Intraoperative imaging and diagnostic challenge

While weaning from CPB immediately after valve implantation, intraoperative 3D TEE (performed in multiple views) revealed apparent immobility of the prosthetic valve’s lateral leaflet. Color Doppler did not indicate mitral stenosis, but the findings raised concern for prosthetic leaflet obstruction (Video 1). The preserved posterior leaflet was suspected of interfering with valve motion, prompting immediate surgical re-exploration.

**Video 1:** Initial intraoperative 3D TEE imaging (midesophageal four-chamber view) showing apparent immobility of the lateral leaflet of the mechanical mitral valve Despite symmetrical prosthesis positioning, the lateral leaflet appears fixed, raising suspicion for a stuck valve. 3D: three-dimensional, TEE: transesophageal echocardiography

Upon reopening the left atrium, no leaflet entrapment, residual chordae, or suture-related obstruction was noted. Manual inspection confirmed symmetrical leaflet motion and function. Although the P2 leaflet measured 15.6 mm on preoperative transthoracic echocardiography (TTE), we excised the P2 segment intraoperatively due to concern that it might intermittently protrude into the path of the mechanical leaflet. Despite this, repeat TEE still demonstrated apparent lateral leaflet immobility (Video 2). A second re-exploration was then performed, and the P3 segment was also excised.

**Video 2:** Repeat intraoperative TEE after first re-exploration still showing apparent lateral leaflet immobility Even after excision of the preserved posterior native leaflet (P2), the same abnormal finding persists, suggesting a possible artifact. TEE: transesophageal echocardiography

To resolve the discrepancy, intraoperative fluoroscopy using a mobile C-arm system in the anteroposterior (AP) view was performed. This revealed normal opening and closing of the mechanical leaflets, with no evidence of restriction or obstruction (Video 3). The echocardiographic findings were therefore attributed to a mirror artifact or reverberation produced by the reflective surfaces of the bileaflet mechanical valve.

**Video 3:** Intraoperative fluoroscopic image (AP view) demonstrating normal bileaflet mechanical valve motion Both leaflets are seen to open and close symmetrically, confirming that the TEE finding was artifactual and that valve function is preserved. AP: anteroposterior, TEE: transesophageal echocardiography

Postoperative course

The patient remained on CPB during both re-explorations and was successfully weaned without further complications. He was extubated on postoperative day 1 and discharged from the ICU on day 7. There were no neurologic or cardiac complications. Postoperative TTE demonstrated a well-functioning mitral prosthesis with a mean pressure gradient of 3 mmHg. The patient was discharged home in stable condition.

## Discussion

TEE plays an important role in intraoperative monitoring during mitral valve surgery, particularly in assessing prosthetic valve function. Although its usage in cardiac surgery has become more common over the years, its limitations must be acknowledged [1]. Especially when assessing mechanical prosthetic valves, TEE artifacts, such as refractions, reverberations, acoustic shadowing, and mirror artifacts, can lead to misinterpretations of prosthetic valve function. In a retrospective study of 170 patients undergoing reoperation for prosthetic valve dysfunction (primarily mitral and aortic valves), Faletra et al. reported a 12% rate of major diagnostic discrepancies between preoperative TEE findings and intraoperative surgical findings, highlighting the potential for misinterpretation even in experienced hands [7].

Echocardiographic artifacts such as refraction, reverberation, acoustic shadowing, and mirror artifacts have been extensively documented in the literature [8,9]. As Bertrand et al. explain, these imaging anomalies are rooted in the basic physics of ultrasound, including reflection off metallic or highly echogenic surfaces and misinterpretation of duplicated structures [6]. Similarly, Faletra et al. showed that 3D TEE, while offering improved spatial resolution, can introduce complex artifacts that challenge interpretation, especially with highly reflective materials such as mechanical prosthetic valves [5,7].

In our case, intraoperative TEE suggested posterior leaflet immobility of a newly implanted mechanical mitral valve. This finding persisted despite surgical re-exploration and excision of the remaining native leaflet tissue. Intraoperative fluoroscopy using a C-arm ultimately demonstrated normal bileaflet valve function, confirming that the TEE findings were artifactual, most likely due to refraction off the valve's reflective surface. To minimize misinterpretation, Bertrand et al. recommend practical strategies: altering imaging planes, adjusting gain, and using complementary imaging modalities. These steps help differentiate true pathology from artifact [6]. This case reaffirms the importance of incorporating multimodal imaging, such as fluoroscopy, when echocardiographic findings are discordant or ambiguous [10]. Clinicians must remain vigilant for artifacts in valve imaging, especially during surgery, where swift and accurate diagnosis is necessary, and use all available tools to avoid unnecessary surgical interventions and associated complications.

## Conclusions

This case highlights the potential for intraoperative echocardiographic artifacts, specifically mirror artifacts, to mimic mechanical valve dysfunction, resulting in unnecessary re-exploration and prolonged CPB time. Extended CPB can increase the risk of postoperative complications, including bleeding, coagulopathy, and organ dysfunction. This underscores the importance of recognizing TEE's limitations, particularly when evaluating mechanical prostheses.

When intraoperative TEE findings are discordant with direct surgical inspection or clinical expectations, it is essential to consider artifact as a differential diagnosis. In such cases, the selective use of alternative imaging modalities, such as intraoperative fluoroscopy, can play a critical role in confirming valve function and avoiding unnecessary surgical interventions. While routine use of fluoroscopy is not recommended, it should be readily considered in ambiguous cases where artifact may drive reintervention decisions.

Improving artifact recognition through structured intraoperative echocardiography training and encouraging real-time collaboration between surgeons and echocardiographers may facilitate earlier identification of imaging pitfalls. Additionally, reviewing multiple TEE planes and utilizing both 2D and 3D modalities can help reduce overreliance on single, artifact-prone views. A systematic, multimodal imaging approach can minimize avoidable surgical risk and improve patient outcomes by accurately distinguishing true pathology from artifact.
